# Competing risk analysis of cardiovascular death in patients with primary gallbladder cancer

**DOI:** 10.1002/cam4.5104

**Published:** 2022-08-03

**Authors:** Chong Chen, Fengshuo Xu, Shiqi Yuan, Xuenuo Zhao, Mengmeng Qiao, Didi Han, Jun Lyu

**Affiliations:** ^1^ Department of Clinical Research The First Affiliated Hospital of Jinan University Guangzhou Guangdong China; ^2^ School of Public Health Shannxi University of Chinese Medicine Xianyang Shaanxi China; ^3^ School of Public Health Xi'an Jiaotong University Health Science Center Xi'an Shaanxi China; ^4^ Department of Neurology The First Affiliated Hospital of Jinan University Guangzhou Guangdong China; ^5^ School of Public Health Qingdao University Qingdao Shangdong China; ^6^ Guangdong Provincial Key Laboratory of Traditional Chinese Medicine Informatization Guangzhou Guangdong China

**Keywords:** cancer education, cancer risk factors, cholangiocarcinoma, clinical cancer research, epidemiology

## Abstract

**Background:**

Developments in medical technology are resulting in continuous decreases in the cancer mortality rate of patients with gallbladder cancer, while non‐cancer deaths in cancer patients are becoming more common. The main cause of this is cardiovascular mortality (CVM). The purpose of this study was to determine the CVM risk in patients with primary gallbladder cancer (PGC).

**Methods:**

We extracted information on patients in the SEER database who were diagnosed with PGC from 2004 to 2015, compared CVM in patients with PGC with the general United States population, and calculated standardized mortality rates (SMRs) and the absolute excess risk. A competing risks model was used to identify and analyze the independent risk factors for cardiovascular death in patients with PGC.

**Results:**

This study included 5925 patients, 247 of whom died from cardiovascular disease. The SMR of cardiovascular death in patients with PGC was 15.84 (95% confidence interval: 15.83–15.85), and the SMR was slightly lower in male than female patients. The competing risks analysis indicated that age, marital status, cancer cell differentiation, chemotherapy status, and year of diagnosis were risk factors for cardiovascular death in patients with PGC.

**Conclusions:**

The CVM risk is considerably higher in patients with PGC than in the general population. It is therefore very necessary to apply cardioprotective interventions to patients with PGC.

## Graphical abstract

Using patient data from the SEER database to assess risk factors for cardiovascular death in Primary gallbladder carcinoma patients, the article adopts a competing risk model approach that can provide a clearer understanding of the link between Primary gallbladder carcinoma and cardiovascular disease. It can better help clinicians make relevant clinical decisions.

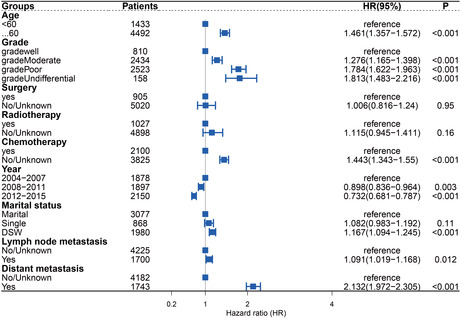

## 1 BACKGROUND

Gallbladder cancer (primary gallbladder cancer [PGC]) is a relatively rare tumor clinically, but it is the most common tumor of the biliary system, accounting for 80%–95% of all tumors of that system.^1^, ^2^ In the United States, nearly 4000 people are diagnosed with PGC each year, and about 2000 die from it.^1^ Because the early stage of the disease is no different from a normal condition, it is often diagnosed in its late stage, and it has a poor prognosis.^3^ With recent improvements in cancer surgery strategies, increased safety awareness of doctors, and changes in daily life, outcomes for patients with PGC have significantly improved.^4^, ^5^ A recent study found that the 5‐year mortality rate of patients with PGC in the United States was about 20.2%.^6^

In 2019, 18.6 million people died from cardiovascular disease (CVD) worldwide, accounting for one‐third of all deaths, and making it the most common cause of mortality.^7^ In the United States, many patients who die of CVD are cancer survivors.^8^

The mortality rate of patients with cancer has recently been decreasing,^9^ and non‐cancer deaths in patients with cancer are also becoming increasingly common, of which CVD is the most common cause.^10^ Cardio‐oncology has recently become a hot topic, with cancers such as colorectal, testicular, and endometrial cancer all being associated with cardiovascular mortality (CVM).^11^, ^12^, ^13^ There is no study that we know of that investigated the relationship between patients with PGC and CVD. We therefore assessed the risk factors for CVD among patients with PGC with the aim of identifying those who require earlier screening and treatment, and to establish preventive measures for CVD in patients with PGC.

## 2 MATERIALS AND METHODS

### 2.1 Data source

The SEER database collects data on cancer diagnoses, treatments, and prognoses for approximately 30% of the United States population, and it is an important population‐based data set that can be used to study the implications of pathological diagnoses across demographics, geographic regions, and time. It is freely available to the public and does not require informed consent from patients or institutional review board approval. It also includes data on chemotherapy and radiotherapy, for which SEER software is licensed.^14^, ^15^

### 2.2 Research variables

**FIGURE 1:**
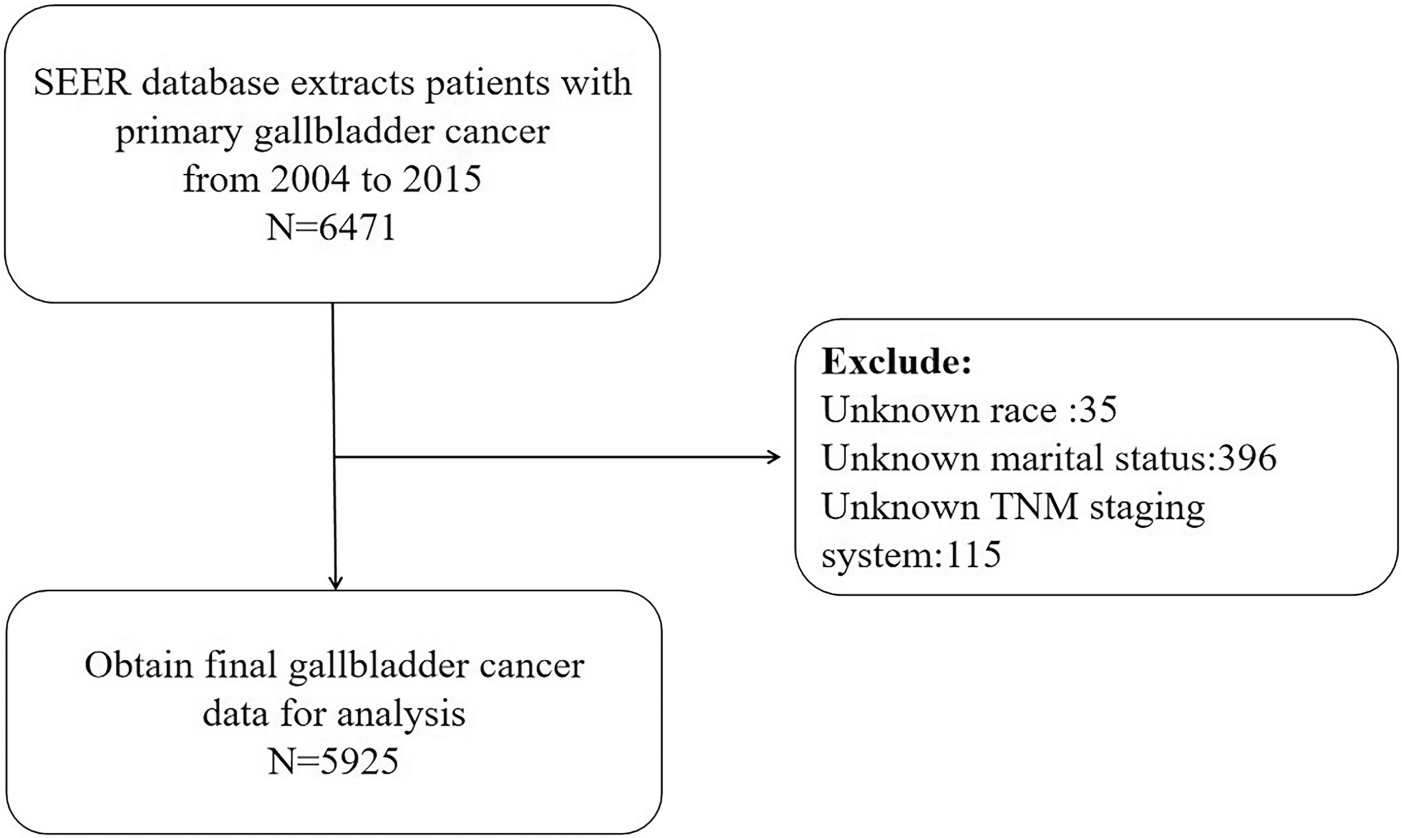
Patient extraction flowchart.

We searched the SEER database for all PGC cases using the ICD‐O‐3 tumor site diagnostic code C23.9, and then obtained information on 5925 patients with PGC older than 18 years according to the inclusion and exclusion criteria (Figure 1). The extracted variables included year of diagnosis, age, sex, ethnicity, marital status, TNM stage, surgery, chemotherapy, and radiotherapy statuses, and cause of death.

The primary outcome of our study was CVD, including the following six causes of death from the SEER database: hypertension without heart disease, cerebrovascular disease, heart disease, atherosclerosis, aortic aneurysm and dissection, and other arterial diseases in the arterioles, capillaries, and vascular system.^12^

### 2.3 Statistical analysis

The article extracted the CVM rate of the general population from 2004 to 2015 from the United States Centers for Disease Control and Prevention, and then compared it with that of patients with PGC to calculate the standardized mortality rate (SMR). Absolute excess risk (AER) was also calculated as (observed death − expected deaths)/(person‐years of observation). The cumulative incidence function was used to estimate the cumulative incidence of time‐point‐specific death, and Nelson‐Aalen estimator curves were plotted for each factor.^16^ Univariate analyses were performed using Gray's test, and factors that were statistically significant in these analyses were then included in the Fine‐Gray subdistribution hazard model in the analysis to identify independent prognostic factors and calculate the hazard ratio (HR) and 95% confidence interval (CI) of each prognostic factor.

The above statistical methods were all implemented using R software, all tests were two‐sided, and the significance threshold was *α* = 0.05.

## 3 RESULTS

### 3.1 Patient characteristics

The information of 6471 patients with PGC was collected after applying the inclusion criteria, and according to the exclusion criteria, we excluded 35 patients without race information, 396 without marital information, and 115 without TNM stage information. Finally, 5925 patients were included in the statistical analysis. The patient details are listed in Table 1, where DSW indicates divorced, separated, or widowed.

**TABLE 1:** Characteristics of the patient cohort.

	*N* = 5925
Age	
<60	1433 (24.2)
≥60	4492 (75.8)
Sex	
Male	1713 (28.9)
Female	4212 (71.1)
Marital status	
Marital	3077 (51.9)
Single	868 (14.6)
DSW	1980 (33.4)
Race	
White	4516 (76.2)
Black	730 (12.3)
Other	679 (11.5)
Year of diagnosis	
2004–2007	1878 (31.7)
2008–2011	1897 (32.0)
2012–2015	2150 (36.3)
Grade	
Well	810 (13.7)
Moderate	2434 (41.1)
Poor	2523 (42.6)
Undifferential	158 (2.7)
Surgery	
Yes	905 (15.3)
No/unknown	5020 (84.7)
Radiotherapy	
Yes	1027 (17.3)
No/unknown	4898 (82.7)
Chemotherapy	
Yes	2100 (35.4)
No/unknown	3825 (64.6)
Lymph node metastasis	
Yes	1700 (28.7)
No/unknown	4225 (71.3)
Distant metastasis	
Yes	1743 (29.4)
No/unknown	4182 (70.6)

### 3.2 SMR and AER

The SMR of cardiovascular death in patients with PGC was 15.84 (95% CI, 15.83–15.84), and the AER was 202.7/10,000. The stratified analysis of the different variables indicated that the SMR and AER of cardiovascular death were higher in patients with PGC. Regarding age stratification, the older the age, the closer the cardiovascular risk of patients with PGC was to that of the general population. For the year of diagnosis, the later the year, the closer the SMR value was to the normal population. Among sexes, females had slightly higher SMRs than males (Table 2).

**TABLE 2:** SMR and AER.

	Observed deaths (%)	Expected deaths	SMR (95% CI)	Excess risk per 10,000	Persons (%)
Total	247	15.59	15.84 (15.83–15.85)	202.66	5925
Male	70	4.53	15.47 (15.45–15.48)	213.86	1713
Female	177	11.03	16.05 (16.03–16.06)	198.60	4212
≤44	2	0.02	81.19 (80.45–81.19)	36.09	226
45–49	2	0.19	10.68 (10.64–10.73)	34.39	240
50–54	7	0.48	14.51 (14.46–14.55)	67.16	377
55–59	10	1.14	8.77 (8.74–8.80)	66.25	590
60–64	14	2.03	6.90 (6.89–6.91)	80.99	694
65–69	16	3.41	4.69 (4.68–4.70)	72.54	784
70–74	28	5.65	4.96 (4.95–4.97)	139.12	803
75–79	42	9.77	4.29 (4.29–4.30)	241.88	804
80–84	46	16.26	2.83 (2.82–2.83)	264.41	732
85+	80	39.45	2.02 (2.02–2.02)	534.45	675
2004–2007	115	5.29	21.73 (21.70–21.75)	263.83	1878
2008–2011	76	4.84	15.68 (15.67–15.70)	185.54	1897
2012–2015	56	5.44	10.29 (10.28–10.30)	147.61	2150
White	200	12.77	15.67 (15.65–15.68)	217.49	4516
Black	26	1.68	15.43 (15.41–15.47)	186.58	730
Other	21	0.67	31.40 (31.27–31.53)	134.96	679

Abbreviations: CI, confidence interval; SMR, standardized mortality rate.

### 3.3 Univariate analyses of cardiovascular death in patients with PGC

**FIGURE 2:**
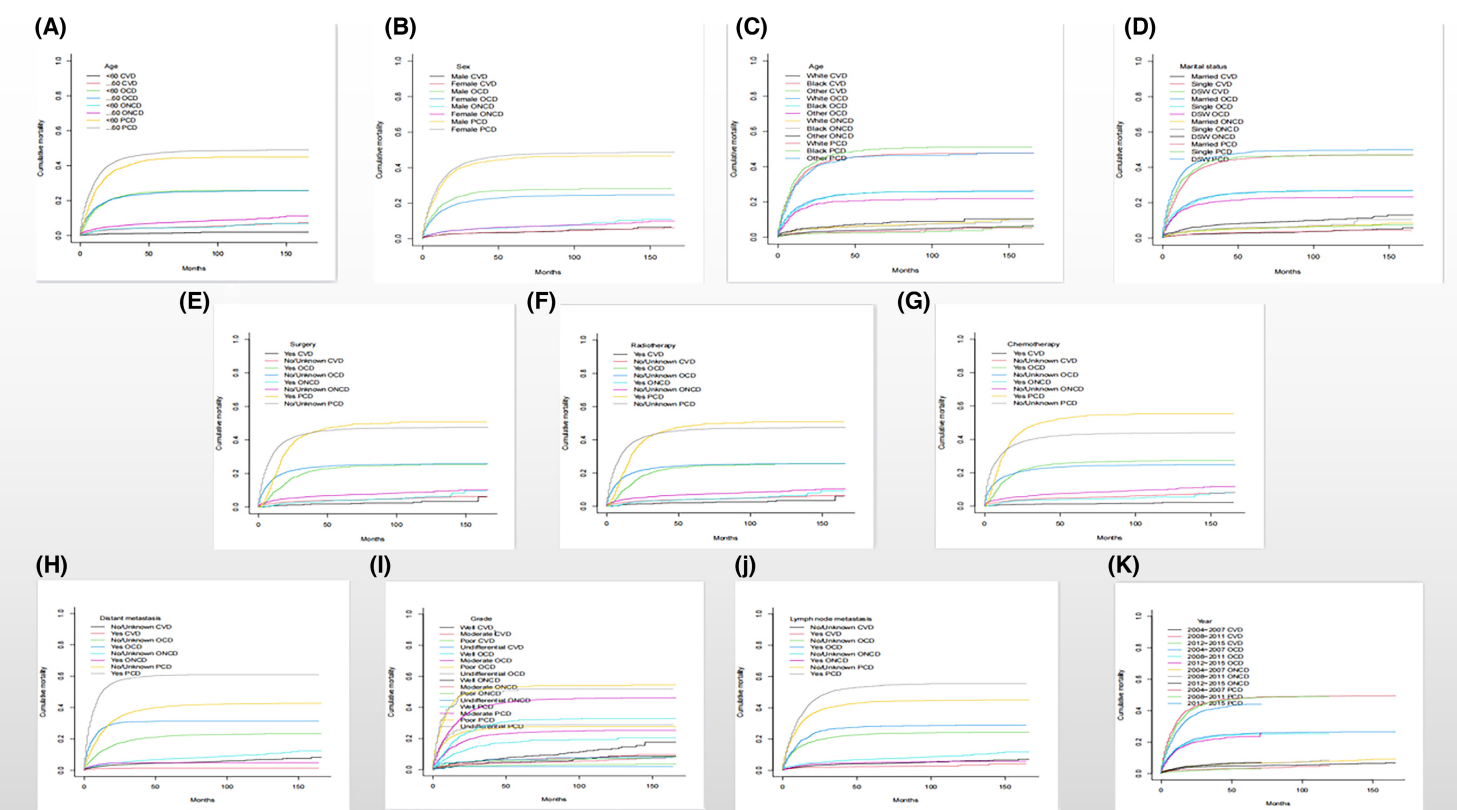
CIF of all causes of death in PGC patients. (A) age, (B) sex, (C) race, (D) marry, (E) surgical, (F) radiotherapy, (G) chemotherapy, (H) distant metastasis, (I) grade, (J) lymph node metastasis, (K) year of diagnosis. CIF, cumulative incidence function; CVD, cardiovascular death; OCDs, other cancer deaths; ONCDs, other non‐cancer deaths; PCD, primary gallbladder cancer death.

**FIGURE 3:**
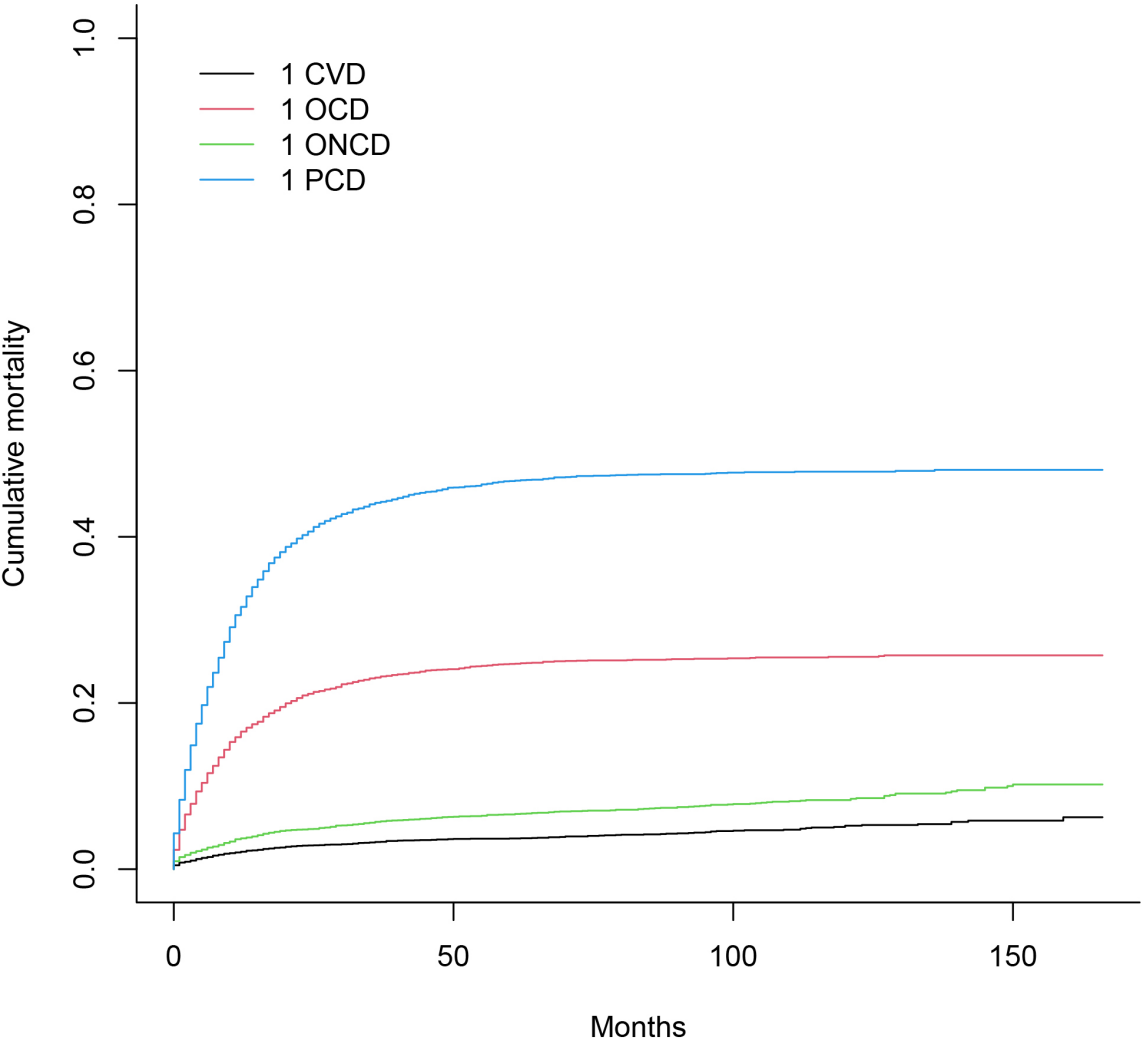
CIF of all subgroups of gallbladder cancer patients dying from all causes. CIF, cumulative incidence function.

Figure 2 displays all causes of death in patients with PGC using the Fine‐Gray subdistribution hazard model. It shows that cardiovascular death had the lowest cumulative mortality, and the cumulative mortality due to primary site death has always been the highest. Figure 3 displays all subgroups of patients with PGC who died from various causes. According to the univariate analysis results, age, marital status, distant metastasis, radiotherapy and chemotherapy statuses, degree of differentiation, year of diagnosis, and lymph node metastasis were associated with cardiovascular death in patients with PGC. Among them, the associations with sex and marital status were not significant.

### 3.4 Multivariate analysis of cardiovascular death in patients with PGC

**FIGURE 4:**
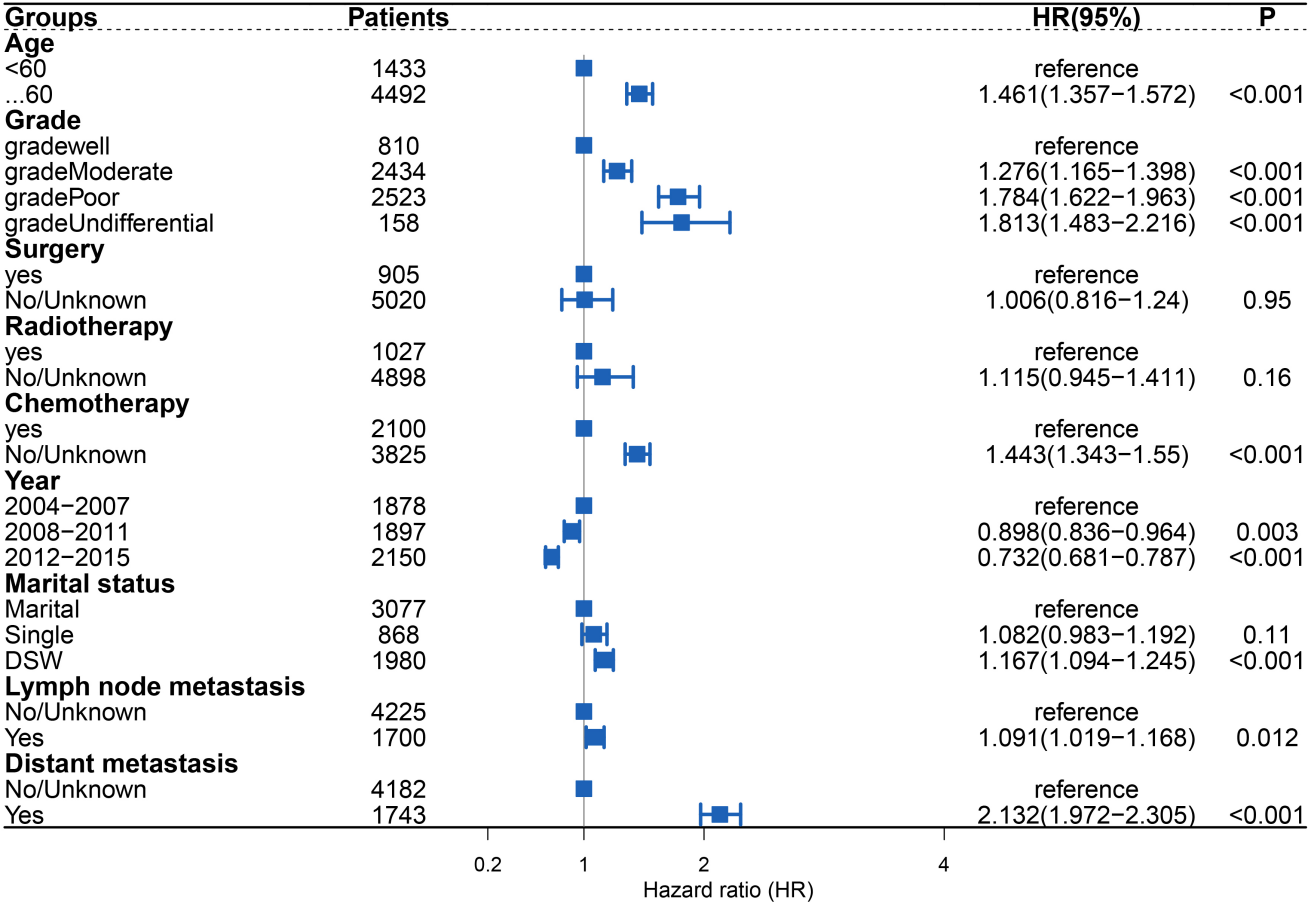
Multifactor competitive risk forest plot.

We included the meaningful variables in the univariate analyses in a multivariate competing risks model, and obtained the following factors with a high risk for cardiovascular death in patients with PGC (Figure 4): age ≥60 years (HR: 1.461; 95% CI: 1.357–1.572), DSW (HR: 1.167; 95% CI: 1.094–1.245), moderately differentiated cancer cells (HR: 1.276; 95% CI: 1.165–1.398), poorly differentiated cancer cells (HR: 1.784; 95% CI: 1.622–1.963), undifferentiated cancer cells (HR: 1.813; 95% CI: 1.483–2.216), cancer cells with lymph node metastasis (HR: 1.091; 95% CI: 1.019–1.168), cancer cells with distant metastasis (HR: 2.132; 95% CI: 1.972–2.305), and no chemotherapy (HR: 1.443; 95% CI: 1.343–1.55). The results also indicated that the later the year of diagnosis, the lower the risk of cardiovascular death (2008–2011 [HR: 0.898; 95% CI: 0.836–0.964]; 2012–2015 [HR: 0.732; 95% CI: 0.681–0.787]).

## 4 DISCUSSION

Cancer and CVD are two common causes of death in humans. Many recent studies have confirmed that the incidence of cardiovascular death is increasing in patients with cancer, and so many governments strongly support cooperation between these two fields in order to better meet the clinical needs of patients with complex cancer.^17^ In a study of 3,234,256 adult patients with cancer,^10^ Sturgeon et al. found that 11% of patients died of CVD, which was far higher proportion than in the general United States population.

Our study found that during 2004–2015, the cardiovascular death rate of patients with PGC in the United States was approximately 4101.7 per 100,000 person‐years, compared with 263.1 per 100,000 person‐years in the general United States population, with an SMR of 15.84 (95% CI, 15.83–15.85). After stratifying by year of diagnosis, we found that the SMR during 2004–2007 was 21.73 (95% CI, 21.70–21.75), and that the SMR became smaller over time, mostly due to population differences.^18^ Regarding age stratification, the SMR continuously decreased as age increased, but generally patients with PGC had a significant advantage over the general population regardless of age group.

We comprehensively assessed the risk of all‐cause death in patients with PGC, and the competing risks analysis revealed that the cumulative mortality rate of cardiovascular death was lower than that of cancer death, other‐cancer death, and other‐cause death. We then incorporated influencing factors identified in the competing risks univariate analyses into a multivariate model, which revealed age at diagnosis, year of diagnosis, marital status, degree of differentiation, chemotherapy status, distant metastasis, and lymph node metastasis as independent factors for cardiovascular death in patients with PGC.

Compared with patients with PGC diagnosed during 2012–2015, those diagnosed during 2004–2007 were more likely to die from CVD, mostly because of advances in medical technology and the emphasis on the convergence of cancer and CVDs.^19^ Most of the patients in the present study were elderly, and the results indicated that the CVM rate was 46% for the elderly patients than for the younger patients. Recent studies have pointed out that the risk factors for cancer and CVD include age,^20^ mostly due to the decline of physical function in elderly patients, various physiological functions not being perfect, and their poor living habits, all of which are associated with CVD occurrence. Being single, divorced, separated, or widowed were also risk factors for cardiovascular death in patients with PGC. Previous studies have found that living alone and social disengagement exert important effects on CVD,^21^ and compared with married patients, single patients may receive less attention and have worse economic, health, and social statuses.^22^, ^23^

Lymph node and distant metastases of cancer cells are present in patients at an advanced stage.^24^ Our study found that lymph node and distant metastases are both risk factors for cardiovascular death in patients with PGC, mostly because cancer cell metastasis can cause symptoms such as inflammation and fever, and also have a greater psychological impact on patients. These can lead to death from CVD.^25^ In addition, the better the degree of differentiation of cancer cells, the lower the likelihood of developing CVD since the cancer cells are closer to normal tissue cells. Data on chemotherapy, radiotherapy, and surgery were included in the competing risks model, which found that patients with PGC who did not receive chemotherapy had a higher risk of CVD. Previous studies have found that existing anticancer drugs such as anthracyclines and trastuzumab are associated with a cumulative heart failure incidence,^26^, ^27^ which was in conflict with our findings, so it is necessary to further verify the relationship between chemotherapy status and patients with PGC. Surgery and radiotherapy are not independent predictors of patients with PGC, and there is a lack of detailed treatment plans for radiotherapy and surgery in the SEER database. Further research on the effects of radiotherapy and surgery on CVM in patients with PGC is therefore needed.

This study had some limitations. First, the SEER database does not guarantee the accuracy of all‐cause death records and may overestimate the probability of cardiovascular death.^28^ Second, the database does not include chemotherapy and radiotherapy doses and other factors associated with CVD. Third, there may have been some minor biases among the study participants due to the retrospective design. Finally, the SEER database lacks certain patient specific information, which inevitably causes some errors to the research ending.

## 5 CONCLUSION

This study found that patients with PGC had a significantly higher risk of CVM than the general United States population, and a competing risks analysis was used to identify independent predictors of CVM in patients with PGC. The age of diagnosis, year of diagnosis, marriage status, degree of differentiation, chemotherapy status, distant metastases, and lymph node metastasis are all independent factor for cardiovascular death in PGC patients. When encountering patients diagnosed with PGC, CVD should be screened as soon as possible, and its risk factors should be controlled.

## AUTHOR CONTRIBUTIONS

Jun Lyu conceptualized the research aims, planned the analyses. Chong Chen and Fengshuo Xu guided the literature review. Shiqi Yuan, Xuenuo Zhao, and Didi Han extracted the data from the SEER database. Shiqi Yuan and Mengmeng Qiao participated in data analysis and interpretation. Chong Chen and Fengshuo Zhao wrote the first draft of the paper and the other authors provided comments and approved the final manuscript.

## FUNDING INFORMATION

The study was supported by Guangdong Provincial Key Laboratory of Traditional Chinese Medicine Informatization (2021B1212040007).

## CONFLICTS OF INTEREST

The authors declare that the research was conducted in the absence of any commercial or financial relationships that could be construed as a potential conflict of interest.

## ETHICS STATEMENT

The data of this study are obtained from the SEER database. The patient's data are public and anonymous, so this study does not require ethical approval and informed consent.

## INFORMED CONSENT STATEMENT

Not applicable.

## PERMISSION TO REPRODUCE MATERIAL FROM OTHER SOURCES

Permit.

## Data Availability

The data that support the findings of this study are openly available in the Surveillance, Epidemiology, and End Results (SEER) database of the National Cancer Institute at https://seer.cancer.gov/.
